# Physical activity based on dance movements as complementary therapy for Parkinson’s disease: Effects on movement, executive functions, depressive symptoms, and quality of life

**DOI:** 10.1371/journal.pone.0281204

**Published:** 2023-02-02

**Authors:** Juliana dos Santos Duarte, Wallesson Amaral Alcantara, Juliana Silva Brito, Livia Cristina Santiago Barbosa, Inara Priscylla Rodrigues Machado, Viviane Kharine Teixeira Furtado, Bruno Lopes dos Santos-Lobato, Denise Silva Pinto, Lane Viana Krejcová, Carlomagno Pacheco Bahia

**Affiliations:** 1 Laboratory of Neuroplasticity, Institute of Health Sciences, Federal University of Pará, Belém, Pará, Brazil; 2 Parkinson Group, Art Science Institute, Federal University of Pará, Belém, Pará, Brazil; 3 Center of Biological and Health Sciences, Federal University of Pará, Belém, Pará, Brazil; Prince Sattam Bin Abdulaziz University, College of Applied Medical Sciences, SAUDI ARABIA

## Abstract

**Background:**

Parkinson’s disease (PD) is a progressive, neurodegenerative disease with motor symptoms that are well understood, but non-motor symptoms may be present and appear at different temporal stages of the disease. Physical activity based on dance movements is emerging as a complementary therapeutic approach to a range of PD symptoms as a multidimensional activity that requires rhythmic synchronization and more neuromuscular functions.

**Objective:**

To evaluate the effects of physical activity based on dance movements on the movement, executive functions, depressive symptoms, quality of life, and severity of PD in individuals diagnosed with PD.

**Methods:**

13 individuals with PD (Hoehn & Yahr I-III, MDS-UPDRS 67.62 ± 20.83), underwent physical activity based on dance movements (2x week for 6 months). Participants were assessed at baseline and after 6 months on movement (POMA, TUG and MDS-UPDRS Part III), executive function (FAB), depressive symptoms (MADRS), quality of life (PDQ-39), and severity of PD (MDS-UPDRS TOTAL). Student’s t-test was used to compare pre and post-intervention results.

**Results:**

We observed a significant improvement in the movement (balance and gait) by the POMA test, *p* = 0.0207, executive function by the FAB test, *p* = 0.0074, abstract reasoning and inhibitory control by the FAB, Conceptualization test, *p* = 0.0062, and Inhibitory Control, *p* = 0.0064, depressive symptoms assessed by the MADRS test significantly reduced, *p* = 0.0214, and the quality of life by the PDQ-39 had a significant increase after the intervention, *p* = 0.0006, showed significant improvements between the pre-and post-intervention periods of physical activity based on dance movements.

**Conclusion:**

Physical activity based on dance movements contributed to significant improvements in movement (balance and gait), executive functions, especially in cognitive flexibility and inhibitory control, and the quality of life too. Sensorimotor integration, most cognitive processing and social skills may have contributed to the results.

**Trial registration:**

The study was registered in the Brazilian registry of clinical trials: RBR-3bhbrb5.

## Introduction

Parkinson’s disease (PD) is the second most frequent neurodegenerative disease in the World and affects around 1% of the global population over 60 years old [1]. It is a systemic disease with a complex clinical manifestation. Beyond the cardinal motor symptoms (bradykinesia, tremor, rigidity, and postural instability), there are non-motor symptoms involving mood disorders, sleep disturbances, cognitive and sensory dysfunctions [2] caused by the neuronal loss in several brain regions together with imbalances in different neurotransmitter systems [3].

The currently available treatments for PD are palliative, do not cover the broad symptomatic spectrum, and do not stop the disease progression [4]. Therefore, non-pharmacologic complementary therapeutic approaches have attracted increasing interest because of their effects on the non-motor symptoms and the quality of life of individuals living with PD.

Physical activity is one of the most widely adopted non-pharmacologic complementary therapeutic approaches for PD, and increasing evidence points to its positive effects on the symptomatic profile [5–9]. In experimental models using small rodents, physical activity reduced risk for the development of PD, improving recovery of motor functions, and neuroprotective effects on dopaminergic neurons [10]. Also, regular physical activity seems to improve neural plasticity through increasing synaptic connections, corticomotor excitation, gray matter volume, and brain-derived neurotrophic factor (BDNF) expression in the human brain [11, 12].

Among the therapeutic approaches based on physical activity such as, for example, physical therapy, aerobic resistance exercises, strength training, and occupational therapy have widely been tested to minimize the progressive development of PD [13–16]. However, more studies are needed to determine the effectiveness of the symptomatic profile [17]. Meanwhile, unconventional approaches such as physical activity based on dance movements, Tai Chi, and virtual reality therapies have been adopted due to specific characteristics that include easy adherence, and compliance, and effects on motor symptoms [18].

There is an increasing interest in the therapeutic applicability of physical activity based on dance movements to the management of neurodegenerative diseases, especially for individuals with PD [19–21]. The physical activity based on dance movements is a multidimensional activity that combines the motor, cognitive, social, emotional, and sensory domains [22]. It may be an excellent way to address motor impairments in individuals with PD through motor stimulation capacities like strength, endurance, flexibility, and functions with neuromuscular demands that include mobility, balance, coordination, and changes in the movement direction [23, 24]. The physical activity based on dance movement demands of cognitive functions through motor learning, memory, creativity, attention, auditory cues, and external sounds rhythmic elements [11, 25, 26]. Additionally, it is considered an enjoyable, motivating, and engaging activity, with good results in adherence for people with PD [27–29].

Therefore, physical activity based on dance movements can be a suitable therapeutic approach to simultaneously address motor and non-motor symptoms of people with PD [30]. Many studies have shown benefits of physical activity based on dance to attenuate symptoms including motor function and quality of life [31]. On the other hand, there are few analyses over non-motor symptoms because the studies are focused on a single aspect of the disease, usually the motor symptoms. Thus, a more throughout evaluation of the beneficial effects of physical activity based on dance movements over the non-motor symptoms of PD need to be explained.

The present study aims to evaluate the effects of physical exercise based on dance movements on the movement, executive functions, depressive symptoms, and quality of life of people with PD. We hypothesize that physical activity based on dance movements may influence the motor and nonmotor symptoms (executive functions, depressive symptoms) and quality of life perception.

## Materials and methods

### Study design

We performed a longitudinal study aiming to analyze the effects of physical activity based on dance movements as a complementary therapy for Parkinson’s disease on the movement, executive functions, depressive symptoms, and quality of life over six months. The participants performed physical activity based on dance movements using the “Baila Parkinson” method [32, 33], and the global effects were assessed by quantitative tests previously validated for people with PD. This study was conducted at the Laboratory of Studies in Functional Rehabilitation (LAERF), Federal University of Pará, in collaboration with the Laboratory of Neuroplasticity at UFPA located in the Belém, Brazil. Ethics proceedings approval of the present study was awarded by the Ethics Committee for Research in Humans from the University Hospital João de Barros Barreto (proc.n.1338241 CEP/HUJBB/UFPA). The study was registered in the Brazilian registry of clinical trials: RBR-3bhbrb5. The participants provided their written informed consent to participate in this study.

### Participants recruitment

The research was developed between May and December 2019. The participants were recruited through social media announcements and subscribed through the research group’s website or telephone. The participants were screened to determine if they met the following criteria: diagnosis according to the UK Parkinson’s disease Society Brain Bank, Hoehn and Yahr stage I to III, under pharmacological treatment for at least 3 years, and on physical conditions to participate in the dance classes. The participants were excluded if they were unable to perform the physical activity based on dance movements, if they had other neurologic or neuropsychiatric conditions, or some comorbidities such as osteoporosis, severe cardiopulmonary diseases, or other conditions that could represent a risk for undergoing physical activities.

### Intervention methods based on dance movements

The participants were submitted to two weekly sessions of physical activity based on dance movements (50 minutes/session), in the afternoon for 6 (six) months. The intervention was delivered in the rooms of laboratory and for small groups of 5 to 6 subjects. The rehabilitation program of exercise based on dance movements followed a protocol based on the combination of different dance styles adapted for appropriateness and safety for individuals with PD, named the “Baila Parkinson” method [32, 33].

The “Baila Parkinson” method consists of progressive movement sequences created by combination and adaptation of choreographic elements from different dance styles executed by people with PD in different stages of the disease. The dance styles included: tango, ballroom dance, urban dances, samba, ballet, contemporary dance, and regional dances. The methodology of dance sessions was adapted to compliance and physical limitation by the people with PD. The sessions were structured around the “work lines” of the method, focused on five aspects of the disease, corresponding to the neuropsychological functions affected by PD: cognitive, sensory, motor, emotional and social aspects [34].

### Outcome measures

First, we evaluated the people with PD before initiation of the physical activity based on dance movements sessions (pre-intervention) and within the week following completion of the six months of attendance to dance therapy sessions (post-intervention), with approximately 50 sessions performed each patient. Demographic data were collected one week before the first intervention session and included age, sex, time since PD diagnosis, and medications. All patients were tested on the day corresponding the half an hour after taking the antiparkinsonian medication, corresponding to the best ON period of the subject. The tests comprised movement, executive functions, depressive symptoms tests, quality of life assessment questionnaire, and **the MDS-UPDRS** to evaluation the general symptomatic presentation (Table 1). Test protocols aimed to evaluate the results of the work lines used in the “Baila Parkinson” dance therapy sessions. We evaluated the movement using the Performance Oriented Mobility Assessment (POMA) test [35] focused on balance and gait. Executive functions were evaluated by the Frontal Assessment Battery (FAB) [36]. We assessed the depressive symptoms by the **Montgomery-Åsberg Depression Rating Scale (MADRS) [37]. The quality of life was assessed using the Parkinson’s Disease Questionnaire– 39 items (PDQ-39) [38], and finally, the severity of Parkinson’s disease was evaluated by the** Movement Disorder Society—Unified Parkinson’s Disease Rating Scale **(MDS-UPDRS TOTAL).**

**Table 1:** Dimensions related to symptomatology and the protocols used to evaluate its results in individuals with PD.

Dimensions	Definition	Assessment Protocols
**Movement**		
Balance and Gait	Fall risk, balance and gait assessment	POMA
**Executive Functions**		
Executive Function	Cognitive flexibility, inhibitory control and working memory	FAB
**Depressive Symptoms**		
Depression		MADRS
**Quality of life**		
Quality of life		PDQ-39
**Symptomatology**		
Assessment of Parkinson’s disease	Severity of Parkinson’s disease	MDS-UPDRS TOTAL

PD: Parkinson Disease; POMA: Performance Oriented Mobility Assessment; FAB: Frontal Assessment Battery; MADRS: Montgomery-Åsberg Depression Rating Scale; PDQ-39: Parkinson’s Disease Questionnaire 39 items; MDS-UPDRS: Movement Disorder Society—Unified Parkinson’s Disease Rating Scale.

### Statistical analysis

We performed statistical analysis using GraphPad Prism® 8.0 Software. First, we tested normality (Kolmogorov-Smirnov) and homogeneity (Levine test) of the data. Then we carried out parametric statistical analyses with a paired t-tests to detect differences between the pre-and post-intervention results. The interval of confidence was set in 95% (p<0,05).

## Results

### Participant characteristics

**Fig 1:**
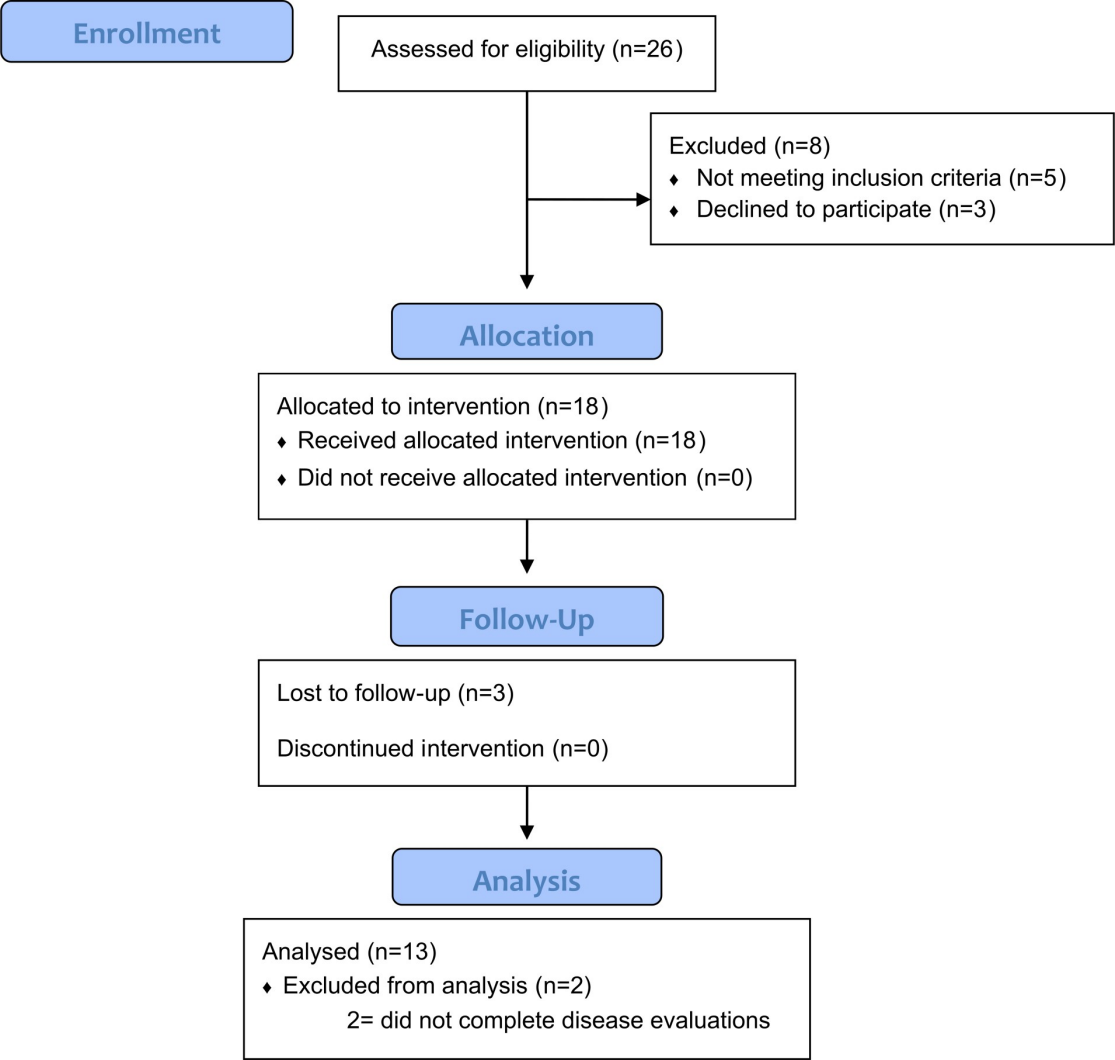
Flowchart of the recruitment of study participants and protocol testing.

Twenty-six individuals with idiopathic PD from which eighteen met the selection criteria and were selected to attend the therapeutic sessions (Fig 1). We had 5 dropouts over the study, and only thirteen of the individuals completed the full period of therapeutic sessions. Therefore, only the thirteen subjects that performed the pre-and post-intervention evaluations were included in the current analysis. Demographic data of the analysed participants are presented in Table 2.

**Table 2:** Demographic and clinical features of participants with PD.

Characteristics (n = 13)	Mean ± SD
Age (years)	65,9 ± 6,5
Gender (female/male)	8F/5M
H&Y	2,2 ± 0,7
Time since PD diagnosis (years)	6,4 ± 3,4
MDS-UPDRS Total Score	67,6 ± 20,8

PD: Parkinson Disease; H&Y: Hoehn and Yahr scale; MDS-UPDRS: Movement Disorder Society—Unified Parkinson’s Disease Rating Scale; SD: Standard deviation.

### Clinical outcomes

#### Movement tests

**Fig 2:**
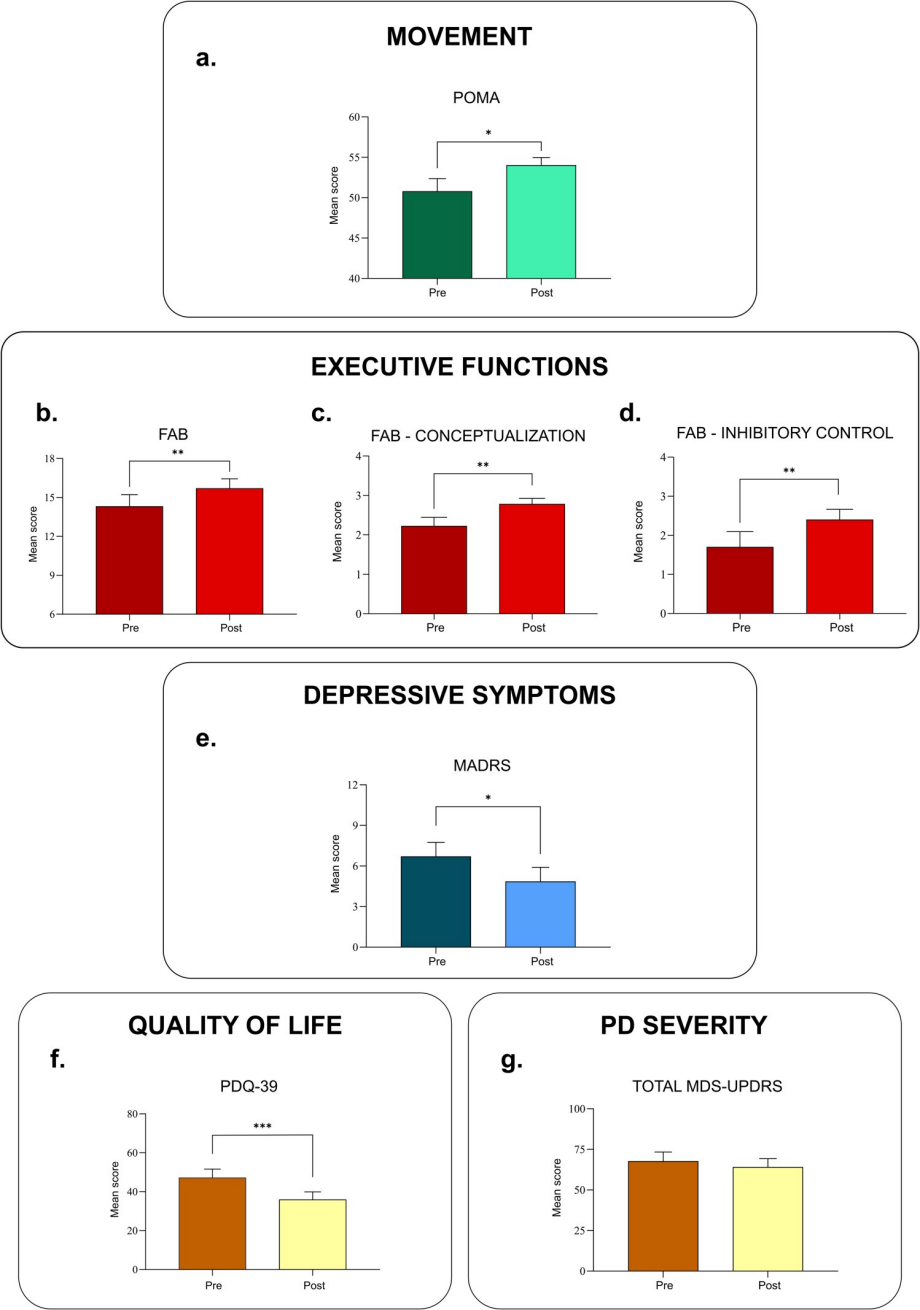
Graphic representations of the mean results obtained during the pre-and post-intervention evaluations for the movement (a), executive functions (b), cognitive flexibility (c), inhibitory control (d), depressive symptoms (e), quality of life (f), and Parkinson’s disease severity (g) assessment protocols. Mean ± S.D. t test, * p<0,05 ** p<0,01 *** p<0,001. POMA: Performance Oriented Mobility Assessment; FAB: Frontal Assessment Battery; MADRS: Montgomery-Åsberg Depression Rating Scale; PDQ-39: Parkinson’s Disease Questionnaire—39 items; MDS-UPDRS: Movement Disorder Society—Unified Parkinson’s Disease Rating Scale.

The POMA scores indicated that there was a significant improvement in the balance and gait of the individuals with PD between the pre-intervention (M = 50.77, SD = 5.70) and post-intervention (M = 54.00, SD = 3.49), *t* (12) = 2.283, *p* = .0207 when submitted to physical activity based on dance movements (Fig 2A). The POMA test results are shown in Table 3.

**Table 3:** Results of individuals with PD pre and post physical activity based on dance movements in the POMA test.

INDIVIDUALS WITH PD	POMA	
	Pre	Post
1°	48	52
2°	51	54
3°	45	44
4°	54	57
5°	50	55
6°	51	57
7°	47	52
8°	37	54
9°	51	55
10°	57	54
11°	55	57
12°	57	57
13°	57	54
**Mean ± SD**	50.77 ± 5.70	54.00 ± 3.49
***p* value**	0,0207*	

PD: Parkinson’s disease; POMA: Performance Oriented Mobility Assessment; SD: Standard deviation.

#### Executive functions test

In the FAB test, the results obtained in the period before the intervention were as follows: total: 14,3 subdomain conceptualization: 2,23±0,59; subdomain inhibitory control: 1,69±1,18. These values were admitted as baseline for the future measurements. We then observed significant improvement in the evaluation of executive functions after physical activity based on dance movements when compared pre-intervention (M = 14.31, SD = 3.28) and post-intervention (M = 15.69, SD = 2.69), *t* (12) = 2.840, *p* = .0074 (Fig 2B). The subdomain of the FAB test, “conceptualization”, indicated a significant improvement in the abstract reasoning between pre-intervention (M = 2.23, SD = 0.59) and post-intervention (M = 2.77, SD = 0.44) when submitted to physical activity based on dance movements, *t* (12) = 2.941, *p* = .0062 (Fig 2C). For the FAB test “inhibitory control” subdomain, the results from the pre-intervention (M = 1.69, SD = 1.18) and post-intervention (M = 2.39, SD = 0.77) indicate that the physical activity based on dance movements resulted in an improvement in inhibitory control, *t* (12) = 2.920, *p* = .0064 (Fig 2D). The FAB test results are shown in Table 4.

**Table 4:** Results of individuals with PD pre and post physical activity based on dance movements in the FAB test.

INDIVIDUALS WITH PD	FAB	
	Pre	Post
1°	5	9
2°	14	16
3°	15	14
4°	17	17
5°	13	15
6°	15	17
7°	18	18
8°	14	16
9°	12	16
10°	14	12
11°	16	18
12°	17	18
13°	16	18
**Mean ± SD**	45,44 ± 14,40	35,65 ± 16,72
***p* value**	0.0074**	

PD: Parkinson’s disease; FAB: Frontal Assessment Battery; SD: Standard deviation.

#### Depressive symptoms tests

The MADRS test showed a significant improvement in the depressive status between pre-intervention (M = 6.69, SD = 3.79) and post-intervention (M = 4.85, SD = 3.82) intervals, *t* (12) = 2.264, *p* = .0214 (Fig 2E). The MADRS test results are shown in Table 5.

**Table 5:** Results of individuals with PD pre and post physical activity based on dance movements in the MADRS test.

INDIVIDUALS WITH PD	MADRS	
	Pre	Post
1°	10	12
2°	11	5
3°	10	6
4°	7	2
5°	2	2
6°	0	0
7°	2	3
8°	7	5
9°	13	12
10°	5	7
11°	7	0
12°	7	5
13°	6	4
**Mean ± SD**	6,69 ± 3,79	4,85 ± 3,83
***p* value**	0,0214*	

PD: Parkinson’s disease; MADRS: Montgomery-Åsberg Depression Rating Scale; SD: Standard deviation.

#### Quality of life questionnaire

The PDQ-39 questionnaire results indicated significant improvement in the quality of life after physical activity based on dance movements when compared pre-intervention (M = 47.19, SD = 16.08) and post-intervention (M = 35.92, SD = 14.40), *t* (12) = 4.239, *p* = .0006 (Fig 2F). The PDQ-39 results are shown in Table 6.

**Table 6:** Results of individuals with PD pre and post physical activity based on dance movements in the PDQ-39 questionnaire.

INDIVIDUALS WITH PD	PDQ-39	
	Pre	Post
1°	51.28	30.13
2°	37.82	29.49
3°	60.90	52.56
4°	31.41	12.82
5°	17.31	9.62
6°	54.49	48.08
7°	42.95	40.38
8°	54.49	57.69
9°	58.33	40.08
10°	66.03	38.46
11°	62.82	42.54
12°	55.77	41.98
13°	19.87	23.12
**Mean ± SD**	47,19 ± 16,08	35,92 ± 14,40
***p* value**	0.0006***	

PD: Parkinson’s disease; PDQ-39: Parkinson’s Disease Questionnaire– 39 items; SD: Standard deviation.

#### Parkinson’s disease severity test

The Total MDS-UPDRS score showed a decrease in PD severity between the pre-intervention (M = 67.62, SD = 20.83) and post-intervention (M = 64.00, SD = 19.13) periods when submitted to physical activity based on dance movements, however these differences were not statistically significant *t* (12) = 0.6002, *p* = .2798 (Fig 2G). The TOTAL MDS-UPDRS results are shown in Table 7.

**Table 7:** Results of individuals with PD pre and post physical activity based on dance movements in the TOTAL MDS-UPDRS.

INDIVIDUALS WITH PD	TOTAL MDS-UPDRS	
	Pre	Post
1°	67	75
2°	47	85
3°	61	60
4°	110	100
5°	85	53
6°	76	74
7°	65	67
8°	68	64
9°	35	40
10°	79	83
11°	83	38
12°	69	41
13°	34	52
**Mean ± SD**	67,62 ± 20,83	64,00 ± 19,13
***p* value**	0.2798	

PD: Parkinson’s disease; TOTAL MDS-UPDRS: Movement Disorder Society—Unified Parkinson’s Disease Rating Scale; SD: Standard deviation.

## Discussion

Thirteen individuals from mild to moderate PD participated in 6 months of physical activity based on dance movements, and our results showed improvements in balance and gait, executive function, depressive symptoms, and quality of life. These improvements corroborate the results of previous studies [30, 39, 40]. The participants reported enjoying classes, and 72.2% of those recruited completed the intervention. Here we have demonstrated that regular attendance to sessions of physical activity based on dance movements can improve the clinical characteristics of PD.

### Movement tests

The POMA test results showed significant improvement in balance and gait after the physical activity based on dance movements. Previous studies have shown improvement in gait and balance but by independent protocols [28, 30, 41–50]. Only the work by Listewnik and Ossowski [39] evaluated balance and gait by using of POMA test in individuals with PD submitted to 12 weeks of dance therapy whereas in our study physical activity based on dance movements was conducted for six months (approximately 24 weeks) in a larger number of subjects. Both studies found significant changes in balance and gait. The POMA test results showed that physical activity based on dance movements significantly reduced the risk of falls in the evaluated subjects. People with PD have a higher incidence of falls due to balance disorders, slower gait, shorter steps, and usually accompanied by freezing [51]. Physical activity based on dance movements has specific characteristics that can attenuate the motor dysfunctions in the PD such as dynamic balance adjustments and spatial perception, visual and auditory cues, use of music, movements in multiple directions, and rhythmic basis [23]. Audiovisual stimuli can perform greater sensory-motor integration and enhance motor control [52].

### Executive functions

Executive functions are the most affected cognitive domain since the early stages of PD [53]. In our study, the participants were in early stages of the disease and the baseline results from FAB tests showed a very mild decline of cognitive executive functions, comparing with results obtained in the same test for people with PD [54]. Some studies have evaluated the effects of dance therapy on the executive functions of individuals with PD and found significant improvement [30, 49]. For the first time, executive functions were evaluated in general and compartmentalized domains to evaluated what executive function specifically showed significant improvement after the physical activity based on dance movements. The results observed may have been achieved from the stimulus provided by our intervention over the executive functions such as cognitive flexibility, inhibitory control, and working memory [55]. For example, new movements and choreographies stimulated cognitive flexibility and inhibitory control by decision-making in movements or choreographic elements not performed impulsively or automatically. Previous studies have shown that dancing reduces the risk of dementia in older people, increases brain white and gray matter volumes, and the concentration of neurotrophic factors compared to other forms of physical exercise [56, 57].

### Depressive symptoms

Depression is the most common neuropsychiatric disorder associated with PD. However, often depressive symptoms are neglected in PD due to the difficulty in identifying them and because they overlap with other symptoms of the disease. The significant reduction in depressive symptoms detected by the MADRS test in our study is in agreement with other studies, although performed by different protocols [30, 58, 59]. Before the intervention, 61.54% of our sample had mild depression by the MADRS test. After six months of physical activity based on dance movements, there was a reduction to 23.08% in the occurrence of mild depressive symptoms. Dancing has positive effects on brain regions such as the anterior cingulate cortex and frontal areas [56], brain structures compromised in depression, which may explain the antidepressant effects in the participants of this study [60]. The perceived individual improvement may be a result of the rhythmic and social characteristics of dance, such as the use of music and group sessions which may be able to modulate serotonergic and dopaminergic systems involved in the regulation of mood and motivation, and alterations in these systems are associated with depression and in patients with PD [60–62]. Study participants reported enjoying the classes, a factor that should also be considered since depressed individuals are less active or have high levels of sedentary behavior [63, 64]. We believe that a more comprehensive, in-depth study of this is needed to build on our results.

### Quality of life

The results showed a significant improvement in the perception of quality of life, which corroborates the results presented in Albany [40]. The concept of quality of life is complex and multifactorial since several factors influence its perception. Manifestation of motor and non-motor symptoms, side effects of treatment [65], subsequent maintenance, interpersonal relationships, financial, and family life characterize the quality of life in PD. Non-motor manifestations such as depression, anxiety, and psychoses are common in individuals with PD and can also worsen the quality of life [66]. Physical activities like dance movements that focus on physical, motor, emotional and social functions can be a key factor in improving quality of life in several domains. The better the quality of life in PD, the greater the chances of this individual to maintain their functional capacity related to independent living. In addition, we assume that the improvement in the perception of quality of life is a result of the perceived improvement in balance and gait, executive functions, and depressive symptoms in individuals with PD.

### Parkinson’s disease severity

Regarding the severity of Parkinson’s disease, our results did not show significant differences in the MDS-UPDRS scores between pre-and post-intervention periods, although other studies have shown the positive results of physical activity based on dance movements approaches over PD progression evaluated by similar tests [30, 40, 44, 67, 68]. One explanation for these results is that, although there are no significant changes in the decrease in PD progression, the fact that our sample did not worsen or remain stable over time is a relevant consideration, as physical activity based on dance movements is an adjuvant approach and reversing or halting disease progression is not intended. Therefore, dance as support to pharmacological treatment is viable, relieving symptoms and improving quality of life.

### Limitations

Important limitations of this study must be considered. The first limitation regards the small sample size and the lack of randomization in the design of this study. The very small sample size leads to wide confidence intervals and imprecision in our estimates. The randomization is missing because i) this is a non-controlled study; and ii) participants were free to make the choice of the activity that was being offered, therefore we cannot rule out selection bias, since people with PD who proactively seek adjuvant therapies for PD symptom mitigation may experience different effects of those who are less active.

Also, the use of PD medications was not monitored during the study. Finally, our sample also showed a high standard deviation of the mean scores in the MDS-UPDRS test, which reflects a high difference in PD severity among the subjects. Nonetheless, this study provides important information regarding the outcomes that are most likely to improve with physical activity based on dance movements for people with PD, and also suggests that a larger, randomized controlled trial is warranted.

## Conclusion

The present study shows some motor and non-motor benefits from physical activity-based in dance for PD’s patients with significant effects on the balance and gait, executive functions, and depressive symptoms that are positive to the quality of life of people with PD. On the other hand, there were no significant changes in functional mobility and PD severity. Characteristic elements of physical activity based on dance movements such as sensorimotor integration, most cognitive processing, and social skills may have contributed to the results obtained in this study. The paradigm we adopted may be effective in future rehabilitation.

## Supporting information

S1 Checklist TREND statement checklist. (PDF)

S1 File (PDF)

S2 File (PDF)

## References

[1] Tysnes O-B, Storstein A. Epidemiology of Parkinson’s disease. Journal of Neural Transmission. 2017;124(8):901–5. doi: 10.1007/s00702-017-1686-y 28150045

[2] Rana AQ, Ahmed US, Chaudry ZM, Vasan S. Parkinson’s disease: a review of non-motor symptoms. Expert review of neurotherapeutics. 2015;15(5):549–62. doi: 10.1586/14737175.2015.1038244 25936847

[3] Moghaddam HS, Zare-Shahabadi A, Rahmani F, Rezaei N. Neurotransmission systems in Parkinson’s disease. Reviews in the Neurosciences. 2017;28(5):509–36. doi: 10.1515/revneuro-2016-0068 28328536

[4] Magrinelli F, Picelli A, Tocco P, Federico A, Roncari L, Smania N, et al. Pathophysiology of Motor Dysfunction in Parkinson’s Disease as the Rationale for Drug Treatment and Rehabilitation. Parkinsons Dis. 2016. 2016. doi: 10.1155/2016/9832839 27366343 PMC4913065

[5] Goodwin VA, Richards SH, Taylor RS, Taylor AH, Campbell JL. The effectiveness of exercise interventions for people with Parkinson’s disease: A systematic review and meta‐analysis. Movement disorders. 2008;23(5):631–40. doi: 10.1002/mds.21922 18181210

[6] Lauzé M, Daneault J-F, Duval C. The effects of physical activity in Parkinson’s disease: a review. Journal of Parkinson’s disease. 2016;6(4):685–98. 10.3233/JPD-160790 PMC5088404 27567884

[7] de Carvalho AO, Sá Filho AS, Murillo-Rodriguez E, Rocha NB, Carta MG, Machado S. Physical exercise for parkinson’s disease: clinical and experimental evidence. Clinical practice and epidemiology in mental health: CP & EMH. 2018;14:89. 29785199 10.2174/1745017901814010089 PMC5897963

[8] Bhalsing KS, Abbas MM, Tan LC. Role of physical activity in Parkinson’s disease. Annals of Indian Academy of Neurology. 2018;21(4):242. doi: 10.4103/aian.AIAN_169_18 30532351 PMC6238554

[9] Fayyaz M, Jaffery SS, Anwer F, Zil-E-Ali A, Anjum I. The effect of physical activity in Parkinson’s disease: a mini-review. Cureus. 2018;10(7). doi: 10.7759/cureus.2995 30245949 PMC6143369

[10] Hou L, Chen W, Liu X, Qiao D, Zhou F-M. Exercise-induced neuroprotection of the nigrostriatal dopamine system in Parkinson’s disease. Frontiers in aging neuroscience. 2017;9:358. doi: 10.3389/fnagi.2017.00358 29163139 PMC5675869

[11] Petzinger GM, Fisher BE, McEwen S, Beeler JA, Walsh JP, Jakowec MW. Exercise-enhanced neuroplasticity targeting motor and cognitive circuitry in Parkinson’s disease. The Lancet Neurology. 2013;12(7):716–26. doi: 10.1016/S1474-4422(13)70123-6 23769598 PMC3690528

[12] Hirsch MA, Iyer SS, Sanjak M. Exercise-induced neuroplasticity in human Parkinson’s disease: what is the evidence telling us? Parkinsonism & related disorders. 2016;22:S78–S81. doi: 10.1016/j.parkreldis.2015.09.030 26439945

[13] Tomlinson CL, Patel S, Meek C, Herd CP, Clarke CE, Stowe R, et al. Physiotherapy intervention in Parkinson’s disease: systematic review and meta-analysis. Bmj. 2012;345. doi: 10.1136/bmj.e5004 22867913 PMC3412755

[14] Uc EY, Doerschug KC, Magnotta V, Dawson JD, Thomsen TR, Kline JN, et al. Phase I/II randomized trial of aerobic exercise in Parkinson disease in a community setting. Neurology. 2014;83(5):413–25. doi: 10.1212/WNL.0000000000000644 24991037 PMC4132568

[15] de Lima TA, Ferreira-Moraes R, Alves WMGdC, Alves TGG, Pimentel CP, Sousa EC, et al. Resistance training reduces depressive symptoms in elderly people with Parkinson disease: A controlled randomized study. Scandinavian Journal of Medicine & Science in Sports. 2019;29(12):1957–67. doi: 10.1111/sms.13528 31357229

[16] Sturkenboom IH, Graff MJ, Hendriks JC, Veenhuizen Y, Munneke M, Bloem BR, et al. Efficacy of occupational therapy for patients with Parkinson’s disease: a randomised controlled trial. The Lancet Neurology. 2014;13(6):557–66. doi: 10.1016/S1474-4422(14)70055-9 24726066

[17] Deane KH, Ellis‐Hill C, Jones D, Whurr R, Ben‐Shlomo Y, Playford ED, et al. Systematic review of paramedical therapies for Parkinson’s disease. Movement disorders: official journal of the Movement Disorder Society. 2002;17(5):984–91. doi: 10.1002/mds.10197 12360547

[18] Alves DRP, McClelland J, Morris M. Complementary physical therapies for movement disorders in Parkinson’s disease: a systematic review. 2015. 26138090

[19] Patterson KK, Wong JS, Prout EC, Brooks D. Dance for the rehabilitation of balance and gait in adults with neurological conditions other than Parkinson’s disease: A systematic review. Heliyon. 2018;4(3):e00584. doi: 10.1016/j.heliyon.2018.e00584 29862347 PMC5968140

[20] Lossing A, Moore M, Zuhl M. Dance as a treatment for neurological disorders. Body, Movement and Dance in Psychotherapy. 2017;12(3):170–84.

[21] Kalyani H, Sullivan K, Moyle G, Brauer S, Jeffrey ER, Roeder L, et al. Effects of dance on gait, cognition, and dual-tasking in Parkinson’s disease: a systematic review and meta-analysis. Journal of Parkinson’s disease. 2019;9(2):335–49. doi: 10.3233/JPD-181516 30958312

[22] McGill A, Houston S, Lee RY. Dance for Parkinson’s: a new framework for research on its physical, mental, emotional, and social benefits. Complementary Therapies in Medicine. 2014;22(3):426–32. doi: 10.1016/j.ctim.2014.03.005 24906580

[23] Earhart GM. Dance as therapy for individuals with Parkinson disease. European journal of physical and rehabilitation medicine. 2009;45(2):231. doi: 10.1016/j.archger.2008.08.006 19532110 PMC2780534

[24] Netz Y. Is there a preferred mode of exercise for cognition enhancement in older age?—a narrative review. Frontiers in medicine. 2019;6:57. doi: 10.3389/fmed.2019.00057 30984760 PMC6450219

[25] de Dreu MJ, Kwakkel G, van Wegen EE. Partnered dancing to improve mobility for people with Parkinson’s disease. Frontiers in neuroscience. 2015;9:444. doi: 10.3389/fnins.2015.00444 26696808 PMC4675848

[26] Dhami P, Moreno S, DeSouza JF. New framework for rehabilitation–fusion of cognitive and physical rehabilitation: the hope for dancing. Frontiers in psychology. 2015;5:1478. doi: 10.3389/fpsyg.2014.01478 25674066 PMC4309167

[27] Shanahan J, Morris ME, Bhriain ON, Saunders J, Clifford AM. Dance for people with Parkinson disease: what is the evidence telling us? Archives of Physical Medicine and Rehabilitation. 2015;96(1):141–53. doi: 10.1016/j.apmr.2014.08.017 25223491

[28] Kunkel D, Fitton C, Roberts L, Pickering R, Roberts H, Wiles R, et al. A randomized controlled feasibility trial exploring partnered ballroom dancing for people with Parkinson’s disease. Clinical Rehabilitation. 2017;31(10):1340–50. doi: 10.1177/0269215517694930 28933613

[29] Michels K, Dubaz O, Hornthal E, Bega D. “Dance therapy” as a psychotherapeutic movement intervention in Parkinson’s disease. Complementary Therapies in Medicine. 2018;40:248–52. doi: 10.1016/j.ctim.2018.07.005 30219460

[30] Hashimoto H, Takabatake S, Miyaguchi H, Nakanishi H, Naitou Y. Effects of dance on motor functions, cognitive functions, and mental symptoms of Parkinson’s disease: a quasi-randomized pilot trial. Complementary therapies in medicine. 2015;23(2):210–9. doi: 10.1016/j.ctim.2015.01.010 25847558

[31] Shanahan J, Morris ME, Ní Bhriain OM, Volpe D, Clifford AM. Dancing and Parkinson’s disease: updates on this creative approach to therapy. 2017.

[32] Krejcova L, Brito J, Cohen W, Bahia C, editors. Impact of Weekly Dance Classes on Quality of Life of Individuals with Parkinson’s Disease. MOVEMENT DISORDERS; 2017: WILEY 111 RIVER ST, HOBOKEN 07030–5774, NJ USA.

[33] Machado IPR, Krejčová LV, Teixeira VK. ALTERAÇÕES NEUROPSIQUIÁTRICAS NA DOENÇA DE PARKINSON: DEPRESSÃO, APATIA E OS EFEITOS DA PRÁTICA DE DANÇA.

[34] Lezak MD, Howieson DB, Loring DW, Fischer JS. Neuropsychological assessment: Oxford University Press, USA; 2004.

[35] Tinetti ME. Performance-oriented assessment of mobility problems in elderly patients. Journal of the American Geriatrics Society. 1986. doi: 10.1111/j.1532-5415.1986.tb05480.x 3944402

[36] Dubois B, Slachevsky A, Litvan I, Pillon B. The FAB: a frontal assessment battery at bedside. Neurology. 2000;55(11):1621–6. doi: 10.1212/wnl.55.11.1621 11113214

[37] Montgomery S, Åsberg M. A new depression scale designed to be sensitive to change: Acad. Department of Psychiatry, Guy’s Hospital; 1977. 10.1192/bjp.134.4.382 444788

[38] Jenkinson C, Fitzpatrick R, Peto V, Greenhall R, Hyman N. The Parkinson’s Disease Questionnaire (PDQ-39): development and validation of a Parkinson’s disease summary index score. Age and ageing. 1997;26(5):353–7. doi: 10.1093/ageing/26.5.353 9351479

[39] Listewnik B, Ossowski ZM. The influence of dance on selected risk factors of falls in Parkinson’s disease patients–A pilot study. Baltic Journal of Health and Physical Activity. 2018;10(1):38–45.

[40] Albani G, Veneziano G, Lunardon C, Vinci C, Daniele A, Cossa F, et al. Feasibility of home exercises to enhance the benefits of tango dancing in people with Parkinson’s disease. Complementary therapies in medicine. 2019;42:233–9. doi: 10.1016/j.ctim.2018.10.028 30670247

[41] Hackney ME, Earhart GM. Health-related quality of life and alternative forms of exercise in Parkinson disease. Parkinsonism & related disorders. 2009;15(9):644–8. doi: 10.1016/j.parkreldis.2009.03.003 19329350 PMC2783812

[42] Hackney ME, Earhart GM. Short duration, intensive tango dancing for Parkinson disease: an uncontrolled pilot study. Complementary therapies in medicine. 2009;17(4):203–7. doi: 10.1016/j.ctim.2008.10.005 19632547 PMC2731655

[43] Volpe D, Signorini M, Marchetto A, Lynch T, Morris ME. A comparison of Irish set dancing and exercises for people with Parkinson’s disease: a phase II feasibility study. BMC geriatrics. 2013;13(1):1–6. doi: 10.1186/1471-2318-13-54 23731986 PMC3685562

[44] McNeely ME, Mai MM, Duncan RP, Earhart GM. Differential effects of tango versus dance for PD in Parkinson disease. Frontiers in Aging Neuroscience. 2015;7:239. doi: 10.3389/fnagi.2015.00239 26733865 PMC4685181

[45] Romenets SR, Anang J, Fereshtehnejad S-M, Pelletier A, Postuma R. Tango for treatment of motor and non-motor manifestations in Parkinson’s disease: a randomized control study. Complementary Therapies in Medicine. 2015;23(2):175–84. doi: 10.1016/j.ctim.2015.01.015 25847555

[46] Ventura MI, Barnes DE, Ross JM, Lanni KE, Sigvardt KA, Disbrow EA. A pilot study to evaluate multi-dimensional effects of dance for people with Parkinson’s disease. Contemporary clinical trials. 2016;51:50–5. doi: 10.1016/j.cct.2016.10.001 27765693 PMC5108673

[47] Sowalsky KL, Sonke J, Altmann LJ, Almeida L, Hass CJ. Biomechanical Analysis of Dance for Parkinson’s Disease: A Paradoxical Case Study of Balance and Gait Effects? EXPLORE. 2017;13(6):409–13. doi: 10.1016/j.explore.2017.03.009 29179887

[48] Shanahan J, Morris ME, Bhriain ON, Volpe D, Lynch T, Clifford AM. Dancing for Parkinson disease: a randomized trial of Irish set dancing compared with usual care. Archives of Physical Medicine and Rehabilitation. 2017;98(9):1744–51. doi: 10.1016/j.apmr.2017.02.017 28336345

[49] de Natale ER, Paulus KS, Aiello E, Sanna B, Manca A, Sotgiu G, et al. Dance therapy improves motor and cognitive functions in patients with Parkinson’s disease. NeuroRehabilitation. 2017;40(1):141–4. doi: 10.3233/NRE-161399 27814308

[50] Allen JL, McKay JL, Sawers A, Hackney ME, Ting LH. Increased neuromuscular consistency in gait and balance after partnered, dance-based rehabilitation in Parkinson’s disease. Journal of Neurophysiology. 2017;118(1):363–73. doi: 10.1152/jn.00813.2016 28381488 PMC5501921

[51] Boonstra TA, van der Kooij H, Munneke M, Bloem BR. Gait disorders and balance disturbances in Parkinson’s disease: clinical update and pathophysiology. Current opinion in neurology. 2008;21(4):461–71. doi: 10.1097/WCO.0b013e328305bdaf 18607208

[52] Azevedo IM, Gondim ITGdO, Silva KMCd, Oliveira CdA, Lins CCdSA, Coriolano MdGWdS. Effects of rhythmic auditory stimulation on functionality in Parkinson’s disease. Fisioterapia em Movimento. 2021;34.

[53] Broeders M, Velseboer DC, de Bie R, Speelman JD, Muslimovic D, Post B, et al. Cognitive change in newly-diagnosed patients with Parkinson’s disease: a 5-year follow-up study. Journal of the International Neuropsychological Society. 2013;19(6):695–708. doi: 10.1017/S1355617713000295 23544964

[54] Lima CF, Meireles LP, Fonseca R, Castro SL, Garrett C. The Frontal Assessment Battery (FAB) in Parkinson’s disease and correlations with formal measures of executive functioning. Journal of neurology. 2008;255(11):1756–61. doi: 10.1007/s00415-008-0024-6 18821046

[55] Diamond A. Executive functions. Annual review of psychology. 2013;64:135–68. doi: 10.1146/annurev-psych-113011-143750 23020641 PMC4084861

[56] Rehfeld K, Lüders A, Hökelmann A, Lessmann V, Kaufmann J, Brigadski T, et al. Dance training is superior to repetitive physical exercise in inducing brain plasticity in the elderly. PloS one. 2018;13(7):e0196636. doi: 10.1371/journal.pone.0196636 29995884 PMC6040685

[57] Verghese J, Lipton RB, Katz MJ, Hall CB, Derby CA, Kuslansky G, et al. Leisure activities and the risk of dementia in the elderly. New England Journal of Medicine. 2003;348(25):2508–16. doi: 10.1056/NEJMoa022252 12815136

[58] Blandy LM, Beevers WA, Fitzmaurice K, Morris ME. Therapeutic argentine tango dancing for people with mild Parkinson’s disease: a feasibility study. Frontiers in neurology. 2015;6:122. doi: 10.3389/fneur.2015.00122 26074873 PMC4445309

[59] Lee N-Y, Lee D-K, Song H-S. Effect of virtual reality dance exercise on the balance, activities of daily living, and depressive disorder status of Parkinson’s disease patients. Journal of physical therapy science. 2015;27(1):145–7. doi: 10.1589/jpts.27.145 25642060 PMC4305547

[60] Gujral S, Aizenstein H, Reynolds CF III, Butters MA, Erickson KI. Exercise effects on depression: possible neural mechanisms. General hospital psychiatry. 2017;49:2–10. doi: 10.1016/j.genhosppsych.2017.04.012 29122145 PMC6437683

[61] Fontanesi C, DeSouza JFX. Beauty That Moves: Dance for Parkinson’s Effects on Affect, Self-Efficacy, Gait Symmetry, and Dual Task Performance. Frontiers in Psychology. 2021;11. doi: 10.3389/fpsyg.2020.600440 33613357 PMC7892443

[62] Aarsland D, Påhlhagen S, Ballard CG, Ehrt U, Svenningsson P. Depression in Parkinson disease—epidemiology, mechanisms and management. Nature Reviews Neurology. 2012;8(1):35–47. 10.1038/nrneurol.2011.189 22198405

[63] Schuch FB, Vancampfort D, Firth J, Rosenbaum S, Ward PB, Silva ES, et al. Physical activity and incident depression: a meta-analysis of prospective cohort studies. American Journal of Psychiatry. 2018;175(7):631–48. doi: 10.1176/appi.ajp.2018.17111194 29690792

[64] Schuch F, Vancampfort D, Firth J, Rosenbaum S, Ward P, Reichert T, et al. Physical activity and sedentary behavior in people with major depressive disorder: a systematic review and meta-analysis. Journal of affective disorders. 2017;210:139–50. doi: 10.1016/j.jad.2016.10.050 28033521

[65] Chaudhuri KR, Odin P, Antonini A, Martinez-Martin P. Parkinson’s disease: the non-motor issues. Parkinsonism & related disorders. 2011;17(10):717–23. doi: 10.1016/j.parkreldis.2011.02.018 21741874

[66] Leroi I, Ahearn DJ, Andrews M, McDonald KR, Byrne EJ, Burns A. Behavioural disorders, disability and quality of life in Parkinson’s disease. Age and ageing. 2011;40(5):614–21. doi: 10.1093/ageing/afr078 21788252

[67] Westheimer O, McRae C, Henchcliffe C, Fesharaki A, Glazman S, Ene H, et al. Dance for PD: a preliminary investigation of effects on motor function and quality of life among persons with Parkinson’s disease (PD). Journal of Neural Transmission. 2015;122(9):1263–70. doi: 10.1007/s00702-015-1380-x 25836752

[68] Solla P, Cugusi L, Bertoli M, Cereatti A, Della Croce U, Pani D, et al. Sardinian Folk Dance for Individuals with Parkinson’s Disease: A Randomized Controlled Pilot Trial. The Journal of Alternative and Complementary Medicine. 2019;25(3):305–16. doi: 10.1089/acm.2018.0413 30624952

