# Supplementary material for: Physical activity based on dance movements as complementary therapy for Parkinson’s disease: Effects on movement, executive functions, depressive symptoms, and quality of life
Source: PLoS One. 2023 Feb 2;18(2):e0281204. doi: 10.1371/journal.pone.0281204 (PMC9894447; doi:10.1371/journal.pone.0281204)
Supplement: S1 File — (PDF) [file pone.0281204.s002.pdf]

Translation of the research project entitled “A dança como terapia adjuvante na reabilitação de pacientes com a doença de Parkinson: ensaios clínicos e metodológicos”

DANCE AS ADJUVANT THERAPY IN THE REHABILITATION OF PATIENTS WITH PARKINSON'S DISEASE: CLINICAL TRIALS AND METHODOLOGICAL

1. STUDY DESIGN

The study will be carried out at the Laboratory of Studies in Functional Rehabilitation (LAERF), at the Institute of Health Sciences of the Federal University of Pará. It will assist elderly neurological patients affected by Parkinson's Disease, arising from spontaneous demand from public calls and continuous flow by registration on an online platform. People over 40 years old diagnosed with Parkinson's Disease and presenting physical conditions to participate in dance activities will form the study intervention group, as evaluated by the responsible neurologist. Volunteers participating in the research will receive dance therapy sessions twice a week, with a duration of 45 to 60 minutes of intervention, to be taught by dance professionals and students of the UFPA Dance Degree course, with application of the dance therapy method. developed aiming at sensorimotor stimulation in the most relevant aspects for symptomatic attenuation in Parkinson's disease, namely the Baila Parkinson method. The intervention will focus on five axes of work: motor, cognitive, emotional, social and body perception. Such intervention will have regular pre-established activities for a period of one year. In order to investigate the effects of the intervention on physiological variables and the symptomatic progression of the disease, we will carry out clinical tests and biochemical analyzes from blood samples when entering the activity and after one year of intervention, in patients undergoing the dance program and in elderly people without a diagnosis of Parkinson's disease or any other neurological disease, matched by age and sex with the experimental group (control group). The data obtained will be organized in a spreadsheet and analyzed using parametric statistics in order to verify the possible differences observed in the motor and non-motor symptoms and biochemical variables of the patients and the control group before and after the intervention period.

2. ABSTRACT

The main objective of this study is to analyze the effects of the regular practice of dance as a non-pharmacological therapeutic tool on the progression of motor and non-motor

symptoms and alteration of biochemical variables in patients with Parkinson's Disease. Parkinson's Disease (PD) is a progressive disorder of the nervous system, which affects the neurons of the mesencephalic substantia nigra, resulting in several motor and non-motor manifestations related to the imbalance of the dopamine neurotransmission system. Dance, for providing new motor possibilities, as well as for the characteristics of socialization and motivation, has great potential for sensorimotor stimulation and can represent an important therapeutic strategy tool, with previous studies demonstrating several physiological benefits and symptomatic attenuation in patients with PD. Patients diagnosed with PD will receive dance therapy sessions using the Baila Parkinson method twice a week for a period of one year. To analyze the effects of weekly dance training on the symptomatic progression and biochemical aspects of these patients, before and after the period we will apply clinical tests, evaluation protocols and we will perform blood collection for analysis. To obtain a control group, elderly people matched by age and sex and without a diagnosis of neurological disease will be submitted to the same analyzes of biochemical parameters and non-specific variables of PD in the evaluation protocols. We will use parametric statistics to analyze possible differences between the groups with PD and control, and between the periods before and after the intervention. The research results will be published in a journal of international circulation, preferably.

3. INTRODUCTION

Traditionally, there is a certain negligence of the biological sciences in relation to the study of the body in relation to dance, even considering that dance is one of the most complex acts that the body can perform, involving from elements of easy description such as the passive locomotor system to elements of profound complexity as the mechanisms involved in the formation of memory, creation and language and the motor act itself. Given its high potential for stimulating neuroplasticity, a growing body of evidence has been raised about its benefits as an adjuvant therapy in the symptomatic attenuation of PD.

PD is a progressive and chronic disorder of the nervous system, which affects several brain regions, contributing to the onset of motor and non-motor symptoms. Its cardinal symptoms are tremor, hypokinesia, rigidity, and balance disorders, and its clinical manifestations result from dopamine deficiency resulting from degeneration of pigmented neurons in the mesencephalic substantia nigra and begins in most patients

between the fifth and sixth decades of life (Mou et al. 2019). The various non-motor symptoms of the disease also affect the quality of life of patients with Parkinson's, involving changes in the cardiovascular, gastrointestinal, urinary, reproductive systems, sleep-wake cycle, mood, memory and attention states, depressive states and anhedonia (Barone et al. 2009).

Late detection of Parkinson's disease tends to be one of the significant limitations in treatment, which is currently still restricted to symptomatic attenuation. Despite advances in understanding the pathophysiology of the disease, diagnosis continues to be made through clinical investigation, based on the onset of motor disorders, whose approach still lacks precision and clinical control (Delenclos et al. 2016). Motor symptoms tend to appear asymmetrically, differently affecting mobility and functionality on both sides of the body (Uitti et al. 2005). Such asymmetry can be observed from the beginning of the disease, and changes during the progression of the disease or in response to treatments (Barrett et al. 2011), making motor assessment of both hands necessary to better identify pathology-related fine motor deficits (San Luciano et al. 2016).

In the biochemical scenario of PD, the death of dopaminergic neurons appears to be the result of a complex interaction of genetic and environmental factors that affect important cellular processes. Many of these processes involve aspects of the immune system associated with aging and dysregulation of inflammatory pathways (Tiwari and Pal 2017). Such inflammatory pathways involve the production of inflammatory cytokines, such as Interleukin 1 (IL-1β), Interleukin 6 (IL-6) and Tumor Necrosis Factor α (TNF-α), whose actions can be neuromodulatory and directly impact the neural networks (Menza et al., 2011).

Several authors have investigated the long-term effect of non-pharmacological therapies for patients with Parkinson's disease (Alves et al. 2015). Physiotherapy is an excellent method to restore and preserve the movement of patients and is widely used (Alves et al. 2015), while dance, which receives more recent attention due to the perception of its therapeutic potential, is one of the modalities of complementary motor therapy capable of inducing therapeutic benefits in PD by improving mobility; body perception and motor coordination and reducing the risk of falls and increasing the quality of life in patients with PD (Heiberger et al. 2011).

Dance can be conceptualized as a sequence of body movements performed in a rhythmic way, accompanied or not by music. Rudolf Laban (Laban, 1990) when

developing his theory of “the art of human movement” states that this art is intrinsically linked to the understanding of its meanings and its relations with the social environment and with the spontaneous acts of human beings, and that the routine of human movements restricts bodily expression. Therefore, the act of dancing can provide new motor possibilities, as well as, due to the characteristics of socialization and motivation, represent an important therapeutic strategy tool. Previous studies using dance as a strategy to maintain or improve motor performance in the elderly have shown that dancing induces several physiological benefits, such as improved cardiorespiratory fitness, physical conditioning, coordination, balance, memorization, rhythm, laterality, motor agility, flexibility, creativity, body awareness and several other elements that improve the general health status and quality of life of the elderly patient (McNeely et al. 2015).

Taking into account the data described here, the present work aims to analyze the effects of weekly dance sessions on motor and non-motor symptoms, quality of life and biochemical aspects in patients with Parkinson's in non-advanced stages. For that, we intend to use the clinical tests "Timed Up-and-Go Test" (TUG), Performance Oriented Balance Assessment (POMA) and the Berg Balance Scale (BBS) associated with the use of protocols of accelerometry to assess motor and balance functions; the frontal assessment battery (FAB), the mental rotation test (MRT) and the CANTAB battery to assess cognitive functions; and the apathy scale (AS) and the Montgomery and Asberg depression scale (MADRS) to investigate the effects of dance on neuropsychiatric aspects, and electroencephalographic recording (EEG) sessions for quantitative electrophysiological analysis. To detect the stage in which the patient is and the progression of the disease, we will use the Hoehn and Yahr scale and the Unified Scale for Parkinson's Disease (UPDRS), respectively (Goetz et al. 2015). In addition, we will perform blood collection in order to investigate possible biochemical changes associated with PD. The concentrations of triglycerides and cholesterol in the blood and the serum levels of inflammatory cytokines will be analyzed, including Interleukin-4, Interleukin-6, Interleukin-10, Interleukin-1β, Tumor Necrosis Factor α, Interferon γ and Tumor Growth Factor. β. Such analyzes will be used as a means of investigating the effects of dance in patients with Parkinson's disease submitted to weekly dance sessions for a period of six months.

4. HYPOTHESES

We work with the hypothesis that the regular practice of dance, as it is characterized in a therapeutic modality that is not restricted only to the aspects of physical exercise, but has the dimensions of pleasantness, stimulation of memory, creation, expression and socialization, must induce improvements in performance in motor and non-motor tests in patients with Parkinson's Disease, as well as positively alter several biochemical markers, helping to reduce the speed of the temporal course of the disease.

5. PRIMARY OBJECTIVE

To study the effects and therapeutic potential of dance in patients with Parkinson's Disease.

6. SECONDARY OBJECTIVE

To analyze the clinical and conceptual relevance of the data, highlighting the importance of using qualitative and quantitative data in the analysis of the benefits of dance as a non-pharmacological strategy to improve the pathological state of patients with Parkinson's.

7. PROPOSED METHODOLOGY

The study will be carried out at the Laboratory of Studies in Functional Rehabilitation (LAERF) at the Institute of Health Sciences of UFPA, with elderly neurological patients affected by Parkinson's Disease, arising from spontaneous demand and who accept to participate in weekly dance classes at no cost to them. Patients to participate in the research will be selected based on the inclusion and exclusion criteria, without the participants having any connection with the research members. The application of clinical tests will be carried out by the neurologist responsible for monitoring them, and by students of the project team coming from courses in the health area, with the need for licenses and training for their application being duly respected. Blood sample collection will be performed by a qualified and trained biomedical doctor, using sterile and appropriate materials and in the environment of the Neuroplasticity Laboratory, ensuring patient safety. A total of 20 milliliters (ml) of blood will be collected from all volunteers after overnight fasting, 10 ml in vacuum tubes with separator gel and 10 ml in vacuum tubes with anticoagulant solution. The serum will be stored at -70°C until transfer to the clinical analysis laboratory at the Federal University of Pará and to the Evandro Chagas Institute, where the biochemical analyzes of the blood samples will be

carried out. The questionnaires and evaluation protocols will be carried out by the research participants, after the patient's clarification about the project and the signing of the Free and Informed Assent Term - TCLE.

Thirty to forty-five people from 40 years of age, of both sexes, diagnosed with Parkinson's Disease and in physical conditions to participate in dance activities, as evaluated by the responsible neurologist, will form the study intervention group. Thirty elderly people without a diagnosis of Parkinson's Disease or any other neurological disease will be invited to compose the control group, matched by age and sex with the experimental group. Elderly people who are not physically able to participate in the dance program will be excluded, either because of an advanced stage of the disease or because of the presentation of comorbidities and other cumulative diseases that pose a risk to the activity, or who do not demonstrate willingness to adhere to the program, or even if they express a desire to discontinue the program during its execution.

Research participants will participate in dance therapy sessions twice a week, lasting 45 to 60 minutes of intervention and applying the Baila Parkinson method, developed by the Parkinson Group at LAERF. The intervention will focus on five axes of work: motor, cognitive, emotional, social and body perception. Such intervention will have regular pre-established dance activities for a period of one year, being regularly taught by students of the Degree in Dance at UFPA and dance teachers graduated from this same course. Data about the presentation of motor and non-motor symptoms will be collected when the participant enters the program, and after one year of intervention, totaling two evaluation batteries between the beginning and the end of the experiments.

The evaluation batteries will consist of performing the clinical tests “Timed Up-and-Go Test” (TUG) (Greene et al. 2010), Performance Oriented Balance Assessment (POMA) (Faber et al. 2006) and the Berg Balance Scale (BBS) (Kornetti et al. 2004) associated with the use of accelerometry protocols to assess motor and balance functions; the Frontal Assessment Battery (FAB) (Dubois et al. 2000) and the Mental Rotation Task (MRT) (Hegarty 2018) to assess cognitive functions; and the Apathy Scale (AS) (Marin et al. 1991) and the Montgomery and Asberg Depression Scale (MADRS) (Snaith et al. 1986) to investigate the effects of dance on neuropsychiatric aspects, and electroencephalographic (EEG) recording sessions) for quantitative electrophysiological analysis of mirror neuron function (Oberman et al. 2005). To detect the stage in which the patient is and the progression of the disease, we will use the Hoehn and Yahr scale and the Unified Scale for Parkinson's Disease (UPDRS),

respectively (Goetz et al. 2015). In addition, we will perform blood collection in order to investigate possible biochemical changes associated with PD. The concentrations of triglycerides and cholesterol in the blood and the serum levels of inflammatory cytokines will be analyzed, including Interleukin-4, Interleukin-6, Interleukin-10, Interleukin-1β, Tumor Necrosis Factor α, Interferon γ and Tumor Growth Factor. β.

Participation in this study is voluntary and will consist of answering the questions contained in the research protocol and participating in the dance program, clinical tests and blood collections. The freedom to withdraw consent and, therefore, the research will be guaranteed at any time. There will also be no personal expenses or compensation for the participant at any stage of the study. This study may present results that will benefit human beings diagnosed with Parkinson's disease, as it may provide information on alternative treatments for neurological deficits and impairments, especially motor and cognitive ones, resulting from Parkinson's disease. In addition, this work will also be able to add, in the scientific community, relevant data on relevant aspects of neuroprotection and neuroinflammation processes in patients with Parkinson's disease and the role of adjuvant therapies on these processes.

8. INCLUSION CRITERIA

The intervention group will include people aged 40 years and over, of both sexes, diagnosed with Parkinson's Disease and in physical condition to participate in dance activities, as assessed by the responsible neurologist. In the control group, elderly people without a diagnosis of Parkinson's disease or any other neurological disease will be included, matched by age and sex with the intervention group.

9. EXCLUSION CRITERIA

People who are not physically able to perform the dance testing and intervention program will be excluded, either because of an advanced stage of the disease or because of the presentation of comorbidities and other cumulative diseases that pose a risk to the activity, or who do not show willingness to adhere to the program, or who express a desire to discontinue the program during its execution.

10. RISKS

The risks involved are ergometric and biological, and involve primarily aspects related to the performance of any physical activity, which includes, for example, risk of falls or

musculoskeletal injuries, and then involve aspects of blood collection, such as venipuncture accidents, with no potential for worsening the patient's neurological condition. In order to minimize such ergometric risks, the students responsible for the execution of the classes in question will be guided and accompanied by the teacher responsible for the project, as well as they are students who have already attended at least one year of the undergraduate course in Dance, and have experience of at least least two years as dance teachers. Also, all students undergo first aid training and pre-hospital care in order to work on the project. Regarding the risks related to blood collection, they are minimized by its exclusive performance by a qualified and trained professional, with experience in the area, in order to reduce the risk of accidents. Regarding the possible embarrassment of the researched in the face of the exposure of the information collected, confidentiality and statistical presentation of the data will be guaranteed to them. In this way, the researchers undertake to fully preserve the anonymity of the identity of the participant registered in the medical record, ensuring the confidentiality of the information provided to the Research Ethics Committee (CEP).

11. BENEFITS

It is assumed that regular participation in the dance therapy program should induce benefits in physical, motor, psychological and cognitive aspects, helping to alleviate symptoms and increase the participants' quality of life.

12. DATA ANALYSIS METHODOLOGY

The data will be organized in a spreadsheet and analyzed through parametric statistics in order to verify the possible differences observed in the motor and non-motor symptoms of the patients during the training period, as well as the differences observed in the quantification of biochemical markers for the experimental groups. In order to detect the significance between the results obtained in the different periods and between the experimental groups, after observing the normality criteria, Tukey's t test and ANOVA analysis of variance will be adopted for the appropriate cases. Spearman's correlation coefficient will be used to verify the correlation between the measurement instruments and the variables of the present study. The significance interval adopted in the study will be at least 95% ($p<0.05$).

13. PRIMARY OUTCOME

Patients participating in the intervention program will likely show improvement/absence of functional decline in clinical motor and non-motor tests, as well as improvement in biochemical tests and perceived quality of life applied during and after the training period.

14. SECONDARY OUTCOME

The quality of life of patients with Parkinson's will show improvements induced by regular dance training as a therapeutic strategy.

15. SAMPLE SIZE IN BRAZIL

Intervention Group: Forty-five people diagnosed with Parkinson's Disease.

Control group: Thirty people without a diagnosis of Parkinson's disease.

16. EXECUTION CRONOGRAM

ACTIVITIES	Months/2020											
	01	02	03	04	05	06	07	08	09	10	11	12
Literature review	X	X										
Selection of research participants	X	X	X	X								
Clinical trials and protocols experimental	X	X									X	X
Dance training		X	X	X	X	X	X	X	X	X	X	X
Data organization										X	X	X
Statistical analysis										X	X	X
Preparation and publication of the research report											X	X

17. REFERENCES

Alves, P. D. R., et al. (2015). Complementary physical therapies for movement disorders in Parkinson's disease: a systematic review.

Barone, P., et al. (2009). "The PRIAMO study: a multicenter assessment of nonmotor symptoms and their impact on quality of life in Parkinson's disease." Movement disorders: official journal of the Movement Disorder Society 24(11): 1641-1649.
Barrett, M. J., et al. (2011). "Handedness and motor symptom asymmetry in Parkinson's disease." Journal of Neurology, Neurosurgery & Psychiatry 82(10): 1122-1124.

Delenclos, M., et al. (2016). "Biomarkers in Parkinson's disease: Advances and strategies." Parkinsonism & related disorders 22: S106-S110.

Dubois, B., et al. (2000). "The FAB: a frontal assessment battery at bedside." Neurology 55(11): 1621-1626.

Faber, M. J., et al. (2006). "Clinimetric properties of the performance-oriented mobility assessment." Physical therapy 86(7): 944-954.

Goetz, C. G., et al. (2015). "Handling missing values in the MDS-UPDRS." Movement Disorders 30(12): 1632-1638.

Greene, B. R., et al. (2010). "Quantitative falls risk assessment using the timed up and go test." IEEE Transactions on Biomedical Engineering 57(12): 2918-2926.

Hegarty, M. (2018). "Ability and sex differences in spatial thinking: What does the mental rotation test really measure?" Psychonomic bulletin & review 25(3): 1212-1219.

Heiberger, L., et al. (2011). "Impact of a weekly dance class on the functional mobility and on the quality of life of individuals with Parkinson’s disease." Frontiers in aging neuroscience 3:14.

Kornetti, D. L., et al. (2004). "Rating scale analysis of the Berg Balance Scale." Archives of physical medicine and rehabilitation 85(7): 1128-1135.

Marin, R. S., et al. (1991). "Reliability and validity of the Apathy Evaluation Scale." Psychiatry research 38(2): 143-162.

McNeely, M., et al. (2015). "Impacts of dance on non-motor symptoms, participation, and quality of life in Parkinson disease and healthy older adults." Maturitas 82(4): 336-341.

Mou, L., et al. (2019). "Open questions on the nature of Parkinson’s disease: from triggers to spreading pathology." Journal of medical genetics: jmedgenet-2019-106210.

Oberman, L. M., et al. (2005). "EEG evidence for mirror neuron dysfunction in autism spectrum disorders." Cognitive brain research 24(2): 190-198.

San Luciano, M., et al. (2016). "Digitized spiral drawing: A possible biomarker for early Parkinson’s disease." PloS one 11(10): e0162799.

Snaith, R., et al. (1986). "Grade Scores of the Montgomery—Åsberg Depression and the Clinical Anxiety Scales." The British journal of psychiatry 148(5): 599-601.

Tiwari, P. C. and R. Pal (2017). "The potential role of neuroinflammation and transcription factors in Parkinson disease." Dialogues in clinical neuroscience 19(1): 71.

Uitti, R. J., et al. (2005). "Parkinson disease: handedness predicts asymmetry." Neurology 64(11): 1925-1930.
