# Supplementary material for: Physical activity based on dance movements as complementary therapy for Parkinson’s disease: Effects on movement, executive functions, depressive symptoms, and quality of life
Source: PLoS One. 2023 Feb 2;18(2):e0281204. doi: 10.1371/journal.pone.0281204 (PMC9894447; doi:10.1371/journal.pone.0281204)
Supplement: S2 File — (PDF) [file pone.0281204.s003.pdf]

A DANÇA COMO TERAPIA ADJUVANTE NA REABILITAÇÃO DE PACIENTES COM A DOENÇA DE PARKINSON: ENSAIOS CLÍNICOS E METODOLÓGICOS.

1. DESENHO DO ESTUDO

O estudo será realizado no Laboratório de Estudos em Reabilitação Funcional (LAERF), no Instituto de Ciências da Saúde da Universidade Federal do Pará. Atenderá pacientes neurológicos idosos acometidos pela Doença de Parkinson, oriundos de demanda espontânea a partir de chamadas públicas e fluxo contínuo por cadastro em plataforma online. Formarão o grupo de intervenção do estudo pessoas a partir de 40 anos diagnosticadas com a Doença de Parkinson e apresentando condições físicas de participarem de atividades de dança, conforme avaliado pelo neurologista responsável. Os voluntários participantes da pesquisa receberão duas vezes por semana sessões de terapia em dança, com duração de 45 a 60 minutos de intervenção, a serem ministradas por profissionais de dança e alunos do curso de Licenciatura em Dança da UFPA, com aplicação de método de dançaterapia desenvolvido objetivando a estimulação sensório-motora nos aspectos mais relevantes para atenuação sintomática na doença de Parkinson, nomeadamente método Baila Parkinson. A intervenção será focada em cinco eixos de trabalho: motor, cognitivo, emocional, social e na percepção corporal. Tal intervenção terá atividades regulares pré-estabelecidas pelo período de um ano. A fim de investigar os efeitos da intervenção sobre variáveis fisiológicas e a progressão sintomática da doença, realizaremos testes clínicos e análises bioquímicas a partir de amostras sanguíneas quando do ingresso na atividade e após um ano de intervenção, nos pacientes submetidos ao programa de dança e em idosos sem diagnóstico de Doença de Parkinson ou qualquer outra doença neurológica, pareados por idade e sexo com o grupo experimental (grupo controle). Os dados obtidos serão organizados em planilha e analisados através de estatística paramétrica a fim de verificar as possíveis diferenças observadas nos sintomas motores e não-motores e variáveis bioquímicas dos pacientes e grupo controle antes e após o período de intervenção.

2. RESUMO

O objetivo principal deste estudo é analisar os efeitos da prática regular da dança como ferramenta terapêutica não-farmacológica sobre a progressão dos sintomas motores e não-motores e alteração de variáveis bioquímicas em pacientes com a Doença de Parkinson. A Doença de Parkinson (DP) é uma desordem progressiva do sistema nervoso, que afeta os neurônios da substância negra mesencefálica resultando em diversas manifestações motoras e não motoras relacionadas ao desbalanço do sistema de neurotransmissão da dopamina. A dança, por proporcionar novas possibilidades motoras, bem como pelas características de sociabilização e motivação, apresenta grande potencial de estimulação sensório-motora e pode representar importante ferramenta de estratégia terapêutica, com estudos anteriores demonstrando diversos benefícios fisiológicos e atenuação sintomática em pacientes com DP. Pacientes diagnosticados com a DP receberão sessões de terapia em dança pelo método Baila Parkinson duas vezes por semana, pelo período de um ano. Para analisar os efeitos do treinamento semanal de dança sobre a progressão sintomática e aspectos bioquímicos desses pacientes, antes e após o período aplicaremos testes clínicos, protocolos de avaliação e realizaremos coleta sanguínea para análises. Para obtenção de grupo controle, idosos pareados por idade e sexo e sem diagnóstico de doença neurológica serão submetidos às mesmas análises

de parâmetros bioquímicos e variáveis não específicas da DP nos protocolos de avaliação. Utilizaremos estatística paramétrica para análise das possíveis diferenças entre os grupos com DP e controle, e entre os períodos anterior e posterior à intervenção. Os resultados da pesquisa serão publicados em periódico de circulação internacional, preferencialmente.

3. INTRODUÇÃO

Tradicionalmente se percebe certa negligência das ciências biológicas em relação ao estudo do corpo com relação à dança, mesmo considerando que a dança é um dos atos mais complexos que o corpo pode realizar, envolvendo desde elementos de fácil descrição como o sistema locomotor passivo até elementos de profunda complexidade como os mecanismos envolvidos na formação de memória, criação e linguagem e do ato motor em si. Dado seu elevado potencial de estimulação da neuroplasticidade, um corpo crescente de evidências vem sendo levantado acerca de seus benefícios enquanto terapia adjuvante na atenuação sintomática da DP.

A DP é uma desordem progressiva e crônica do sistema nervoso, que afeta diversas regiões encefálicas, contribuindo para o aparecimento de sintomas motores e não motores. Tem como sintomas cardinais: tremor, hipocinesia, rigidez e distúrbios do equilíbrio, e suas manifestações clínicas decorrem da deficiência de dopamina resultante de degeneração dos neurônios pigmentados da substância negra mesencefálica e inicia na maioria dos pacientes entre a quinta e a sexta décadas de vida (Mou et al. 2019). Os diversos sintomas não-motores da doença também afetam a qualidade de vida do paciente com Parkinson, envolvendo alterações nos sistemas cardiovascular, gastrointestinal, urinário, reprodutivo, ciclo sono-vigília, estados de humor, memória e atenção, estados depressivos e anedonia (Barone et al. 2009).

A detecção tardia da doença de Parkinson tende a ser uma das limitações significativas no tratamento, que atualmente ainda é restrito à atenuação sintomática. Apesar dos avanços na compreensão da fisiopatologia da doença, o diagnóstico continua sendo feito através da investigação clínica, baseado no aparecimento dos distúrbios motores, cuja abordagem ainda carece de precisão e controle clínico (Delenclos et al. 2016). Os sintomas motores tendem a aparecer de forma assimétrica, afetando de maneira distinta a mobilidade e a funcionalidade dos dois lados do corpo (Uitti et al. 2005). Tal assimetria pode ser observada desde o início da doença, e sofre alterações durante a progressão da mesma ou em resposta a tratamentos (Barrett et al. 2011) tornando necessária a avaliação motora de ambas as mãos para melhor identificar déficits motores finos relacionados à patologia (San Luciano et al. 2016).

No cenário bioquímico da DP, a morte de neurônios dopaminérgicos parece ser resultado de uma complexa interação de fatores genéticos e ambientais que afetam processos celulares importantes. Muitos desses processos envolvem aspectos do sistema imune associados ao envelhecimento e desregulação das vias inflamatórias (Tiwari e Pal 2017). Tais vias inflamatórias envolvem a produção de citocinas inflamatórias, como a Interleucina 1 (IL-1β), Interleucina 6 (IL-6) e o Fator de Necrose Tumoral α (TNF-α),cujas ações podem ser neuromodulatórias e impactar diretamente os processos neurais (Menza et al., 2011).

Diversos autores tem investigado o efeito em longo prazo de terapias não-farmacológicas para pacientes com a doença de Parkinson (Alves et al. 2015). A fisioterapia constitui excelente método para restaurar e preservar a movimentação dos pacientes sendo amplamente utilizada

(Alves et al. 2015), enquanto a dança, que recebe atenção mais recente devido à percepção de seu potencial terapêutico, consiste em uma das modalidades de terapia motora complementar capaz de induzir benefícios terapêuticos na DP melhorando a mobilidade; a percepção corporal e a coordenação motora e reduzindo o risco de quedas e aumentando a qualidade de vida em pacientes com DP (Heiberger et al. 2011).

A dança pode ser conceituada como uma sequência de movimentos corporais executados de maneira ritmada, acompanhados ou não de música. Rudolf Laban (Laban, 1990) ao desenvolver a sua teoria "da arte do movimento humano" afirma que esta arte é intrinsecamente ligada à compreensão dos seus significados e de suas relações com o meio social e com os atos espontâneos dos seres humanos, e que a rotina dos movimentos humanos restringe a expressão corporal. Assim sendo, o ato de dançar pode proporcionar novas possibilidades motoras, bem como, pelas características de socialização e motivação, representar como uma importante ferramenta de estratégia terapêutica. Estudos anteriores utilizando a dança como estratégia de manutenção ou melhora no desempenho motor em idosos têm demonstrado que dançar induz diversos benefícios fisiológicos, como melhora da aptidão cardiorrespiratória, condicionamento físico, coordenação equilíbrio, memorização, ritmo, lateralidade, agilidade motora, flexibilidade, criatividade, consciência corporal e diversos outros elementos que melhoram o estado geral de saúde e a qualidade de vida do paciente idoso (McNeely et al. 2015).

Tomando em nota os dados aqui descritos, o presente trabalho se propõe a analisar os efeitos de sessões semanais de dança sobre os sintomas motores, não motores, a qualidade de vida e aspectos bioquímicos em pacientes com Parkinson em estágios não avançados. Para tanto, pretendemos utilizar os testes clínicos "Timed Up-and-Go Test" (TUG), Avaliação do Equilíbrio Orientada pelo Desempenho (POMA) e a Escala de Equilíbrio de Berg (Berg Balance Scale – BBS) associados ao uso de protocolos de acelerometria para avaliar as funções motoras e de equilíbrio; a bateria de avaliação frontal (Frontal Assessment Battery – FAB), o teste de rotação mental (Mental Rotation Task – MRT) e bateria CANTAB para avaliar as funções cognitivas; e a escala de apatia (AS) e a escala de depressão Montgomery e Asberg (MADRS) para investigar os efeitos da dança em aspectos neuropsiquiátricos, e sessões de registro eletroencefalográfico (EEG) para análise eletrofisiológica quantitativa. Para detectar o estágio no qual o paciente encontra-se e a progressão da doença utilizaremos a escala de Hoehn e Yahr e a Escala Unificada para a doença de Parkinson (UPDRS), respectivamente (Goetz et al. 2015). Além disso, realizaremos coleta sanguínea com o intuito de investigar as possíveis alterações bioquímicas associadas à DP. Serão analisadas as concentrações de triglicerídeos e colesterol no sangue e os níveis séricos de citocinas inflamatórias, dentre elas a Interleucina-4, Interleucina-6, Interleucina-10, Interleucina-1β, Fator de Necrose Tumoral α, Interferon γ e Fator de Crescimento Tumoral β. Tais análises serão utilizadas como meio de investigação sobre os efeitos da dança em pacientes com a doença de Parkinson submetidos a sessões semanais em dança por um período de seis meses.

4. HIPÓTESE

Trabalhamos com a hipótese que a prática regular de dança, por se caracterizar em uma modalidade terapêutica que não se restringe apenas aos aspectos do exercício físico, mas tem as dimensões da aprazibilidade, estimulação da memória, da criação, da expressão e da

socialização, há de induzir melhoras no desempenho nos testes motores e não-motores nos pacientes com a Doença de Parkinson, bem como alterar positivamente diversos marcadores bioquímicos, auxiliando a reduzir a velocidade do curso temporal da doença.

5. OBJETIVO PRIMÁRIO

Estudar os efeitos e potencial terapêutico da dança em pacientes com a Doença de Parkinson.

6. OBJETIVO SECUNDÁRIO

Analisar a relevância clínica e conceitual dos dados realçando a importância do uso de dados qualitativos e quantitativos na análise dos benefícios da dança como estratégia não-farmacológica de melhora do estado patológico de pacientes com Parkinson.

7. METODOLOGIA PROPOSTA

O estudo será realizado no Laboratório de Estudos em Reabilitação Funcional (LAERF) no Instituto de Ciências da Saúde da UFPA, com pacientes neurológicos idosos acometidos pela Doença de Parkinson, oriundos de demanda espontânea e que aceitem participar de aulas semanais de dança sem quaisquer ônus para eles. Os pacientes para participarem da pesquisa serão selecionados com base nos critérios de inclusão e exclusão, sem que os participantes tenham qualquer vínculo com os membros da pesquisa. A aplicação dos testes clínicos se dará pelo neurologista responsável pelo acompanhamento dos mesmos, e por discentes da equipe do projeto oriundos de cursos na área da saúde, sendo devidamente respeitada a necessidade de licenças e treinamento para a aplicação dos mesmos. A coleta de amostra sanguínea será realizada por um biomédico qualificado e treinado, com a utilização de materiais estéreis e adequados e no ambiente do Laboratório de Neuroplasticidade, garantindo a segurança do paciente. Serão coletados um total de 20 mililitros (ml) de sangue de todos os voluntários após jejum noturno, 10 ml em tubos a vácuo com gel separador e 10 ml em tubos a vácuo com solução anticoagulante. O soro será armazenado a -70°C até a transferência para o laboratório de análises clínicas da Universidade Federal do Pará e para o Instituto Evandro Chagas, onde serão realizadas as análises bioquímicas das amostras sanguíneas. Os questionários e protocolos de avaliação serão realizados pelos participantes da pesquisa, após o esclarecimento do paciente acerca do projeto e a assinatura do Termo de Assentimento Livre e Esclarecido – TCLE.

Formarão o grupo de intervenção do estudo trinta a quarenta e cinco pessoas a partir de 40 anos, de ambos os sexos, diagnosticados com a Doença de Parkinson e em condições físicas de participarem das atividades de dança, conforme avaliado pelo neurologista responsável. Para compor o grupo controle serão convidados trinta idosos sem diagnóstico de Doença de Parkinson ou qualquer outra doença neurológica, pareados por idade e sexo com o grupo experimental. Serão excluídos os idosos que não apresentarem condições físicas de realizarem o programa de dança, seja por estágio avançado da doença seja por apresentação de quadro de comorbidades e outras doenças cumulativas que imponham risco para a atividade, ou que não

demonstrarem vontade de aderir ao programa, ou ainda que exprimam desejo de descontinuar o programa durante sua execução.

Os participantes da pesquisa participarão de sessões de terapia em dança duas vezes por semana, com duração de 45 a 60 minutos de intervenção e com aplicação do método Baila Parkinson, desenvolvido pelo Grupo Parkinson no LAERF. A intervenção será focada em cinco eixos de trabalho: motor, cognitivo, emocional, social e na percepção corporal. Tal intervenção terá atividades regulares pré-estabelecidas de dança por um período de um ano, sendo ministrado regularmente por discentes do curso de Licenciatura em Dança da UFPA e professores de dança graduados por este mesmo curso. Os dados acerca da apresentação dos sintomas motores e não-motores serão coletados quando do ingresso do participante no programa, e após um ano de intervenção, totalizando duas baterias de avaliação entre o início e o final dos experimentos.

As baterias de avaliação consistirão na realização dos testes clínicos “Timed Up-and-Go Test” (TUG) (Greene et al. 2010), Avaliação do Equilíbrio Orientada pelo Desempenho (POMA) (Faber et al. 2006) e a Escala de Equilíbrio de Berg (Berg Balance Scale – BBS) (Kornetti et al. 2004) associados ao uso de protocolos de acelerometria para avaliar as funções motoras e de equilíbrio; a bateria de avaliação frontal (Frontal Assessment Battery – FAB) (Dubois et al. 2000) e o teste de rotação mental (Mental Rotation Task – MRT) (Hegarty 2018) para avaliar as funções cognitivas; e a escala de apatia (AS) (Marin et al. 1991) e a escala de depressão Montgomery e Asberg (MADRS) (Snaith et al. 1986) para investigar os efeitos da dança em aspectos neuropsiquiátricos, e sessões de registro eletroencefalográfico (EEG) para análise eletrofisiológica quantitativa da função dos neurônios espelho (Oberman et al. 2005). Para detectar o estágio no qual o paciente encontra-se e a progressão da doença utilizaremos a escala de Hoehn e Yahr e a Escala Unificada para a doença de Parkinson (UPDRS), respectivamente (Goetz et al. 2015). Além disso, realizaremos coleta sanguínea com o intuito de investigar as possíveis alterações bioquímicas associadas à DP. Serão analisadas as concentrações de triglicerídeos e colesterol no sangue e os níveis séricos de citocinas inflamatórias, dentre elas a Interleucina-4, Interleucina-6, Interleucina-10, Interleucina-1β, Fator de Necrose Tumoral α, Interferon γ e Fator de Crescimento Tumoral β.

A participação neste estudo é voluntária e consistirá em responder as perguntas contidas no protocolo de pesquisa e a participar do programa de dança, dos testes clínicos e das coletas sanguíneas. Será garantida a liberdade da retirada do consentimento e, por conseguinte da pesquisa a qualquer momento. Também não existirão despesas ou compensações pessoais para o participante em qualquer fase do estudo. Este estudo pode apresentar resultados que beneficiarão seres humanos com diagnóstico de doença de Parkinson, pois poderá fornecer informações sobre tratamentos alternativos para os déficits e comprometimentos neurológicos, especialmente motores e cognitivos, decorrentes da doença de Parkinson. Além disso, este trabalho também poderá acrescentar, na comunidade científica, dados relevantes sobre aséctos relevantes dos processos de neuroproteção e neuroinflamação em pacientes com doença de Parkinson e a função das terapias adjuvantes sobre esses processos.

8. CRITÉRIOS DE INCLUSÃO

No grupo de intervenção serão inclusas as pessoas a partir de 40 anos, de ambos os sexos, diagnosticados com a Doença de Parkinson e em condições físicas de participarem das atividades de dança, conforme avaliado pelo neurologista responsável.

No grupo controle, serão inclusos idosos sem diagnóstico de Doença de Parkinson ou qualquer outra doença neurológica, pareados por idade e sexo com o grupo de intervenção.

9. CRITÉRIOS DE EXCLUSÃO

Serão excluídas as pessoas que não apresentarem condições físicas de realizarem o programa de testes e de intervenção com dança, seja por estágio avançado da doença seja por apresentação de quadro de comorbidades e outras doenças cumulativas que imponham risco para a atividade, ou que não demonstrarem vontade de aderir ao programa, ou ainda que exprimam desejo de descontinuar o programa durante sua execução.

10. RISCOS

Os riscos envolvidos são ergométricos e biológicos, e envolvendo primeiramente aspectos relacionados à realização de qualquer atividade física, que inclui por exemplo risco de quedas ou lesões musculoesqueléticas, e em seguida envolvem aspectos da coleta sanguínea, como acidentes de punção venosa, não apresentando potencial de agravo à condição neurológica do paciente. A fim de minimizar tais riscos ergométricos, os alunos responsáveis pela execução das aulas em questão serão orientados e acompanhados pela professora responsável pelo projeto, bem como são alunos que já cursaram pelo menos um ano do curso de graduação em Dança, e possuem experiência de pelo menos dois anos como professores de dança. Ainda, todos os alunos passam por treinamento de primeiros socorros e atendimento pré-hospitalar para poderem atuar no projeto. Com relação aos riscos relacionados à coleta sanguínea, minimizam-se pela sua exclusiva realização por um profissional qualificado e treinado, com experiência na área, com o intuito de reduzir os riscos de acidentes. Quanto ao possível constrangimento do pesquisado frente à exposição das informações coletadas, será garantida, a estes, a confidencialidade e a apresentação estatística dos dados. Desta maneira, os pesquisadores se comprometem a preservar integralmente o anonimato da identidade do participante registrado no prontuário, assegurando o sigilo das informações prestadas ao Comitê da Ética em Pesquisa (CEP).

11. BENEFÍCIOS

Assume-se que a participação regular no programa de terapia em dança deverá induzir benefícios nos aspectos físicos, motores, psicológicos e cognitivos auxiliando na atenuação sintomática e aumentando a qualidade de vida dos participantes.

12. METODOLOGIA DE ANÁLISE DE DADOS

Os dados serão organizados em planilha e analisados através de estatística paramétrica a fim de verificar as possíveis diferenças observadas nos sintomas motores e não-motores dos pacientes durante o período de treino bem como, as diferenças observadas nas quantificações dos marcadores bioquímicos para os grupos experimentais. A fim de detectar a significância entre os resultados obtidos nos diferentes períodos e entre os grupos experimentais, após observados os critérios de normalidade, serão adotados o Teste t de Tukey e a análise de variância ANOVA para os casos que couber. O coeficiente de correlação de Spearman será utilizado para verificar a correlação entre os instrumentos de medida e as variáveis do presente estudo. O intervalo de significância adotado no estudo será de no mínimo de 95% ($p<0.05$).

13. DESFECHO PRIMÁRIO

Os pacientes participantes do programa de intervenção provavelmente apresentarão melhora/ausência de declínio funcional nos testes clínicos motores e não motores, bem como melhora nos testes bioquímicos e percepção de qualidade de vida aplicados durante e após o período de treinamento.

14. DESFECHO SECUNDÁRIO

A qualidade de vida dos pacientes com Parkinson apresentará melhoras induzidas pelo treinamento regular em dança como estratégia terapêutica.

15. TAMANHO DA AMOSTRA NO BRASIL

Grupo Intervenção: Quarenta e cinco pessoas diagnosticadas com a Doença de Parkinson.

Grupo controle: Trinta pessoas sem diagnóstico de Doença de Parkinson.

16. CRONOGRAMA DE EXECUÇÃO

ATIVIDADES	Meses/2020											
	01	02	03	04	05	06	07	08	09	10	11	12
Revisão bilbiográfica	X	X										
Seleção dos participantes da pesquisa	X	X	X	X								
Testes clínicos e protocolos experimentais	X	X									X	X
Treinamento em dança		X	X	X	X	X	X	X	X	X	X	X

Organização dos dados										X	X	X
Análises estatísticas										X	X	X
Elaboração e publicação do relatório da pesquisa											X	X

17. REFERÊNCIAS BIBLIOGRÁFICAS

Alves, P. D. R., et al. (2015). Complementary physical therapies for movement disorders in Parkinson's disease: a systematic review.

Barone, P., et al. (2009). "The PRIAMO study: a multicenter assessment of nonmotor symptoms and their impact on quality of life in Parkinson's disease." Movement disorders: official journal of the Movement Disorder Society 24(11): 1641-1649.

Barrett, M. J., et al. (2011). "Handedness and motor symptom asymmetry in Parkinson's disease." Journal of Neurology, Neurosurgery & Psychiatry 82(10): 1122-1124.

Delenclos, M., et al. (2016). "Biomarkers in Parkinson's disease: Advances and strategies." Parkinsonism & related disorders 22: S106-S110.

Dubois, B., et al. (2000). "The FAB: a frontal assessment battery at bedside." Neurology 55(11): 1621-1626.

Faber, M. J., et al. (2006). "Clinimetric properties of the performance-oriented mobility assessment." Physical therapy 86(7): 944-954.

Goetz, C. G., et al. (2015). "Handling missing values in the MDS-UPDRS." Movement Disorders 30(12): 1632-1638.

Greene, B. R., et al. (2010). "Quantitative falls risk assessment using the timed up and go test." IEEE Transactions on Biomedical Engineering 57(12): 2918-2926.

Hegarty, M. (2018). "Ability and sex differences in spatial thinking: What does the mental rotation test really measure?" Psychonomic bulletin & review 25(3): 1212-1219.

Heiberger, L., et al. (2011). "Impact of a weekly dance class on the functional mobility and on the quality of life of individuals with Parkinson’s disease." Frontiers in aging neuroscience 3: 14.

Kornetti, D. L., et al. (2004). "Rating scale analysis of the Berg Balance Scale." Archives of physical medicine and rehabilitation 85(7): 1128-1135.

Marin, R. S., et al. (1991). "Reliability and validity of the Apathy Evaluation Scale." Psychiatry research 38(2): 143-162.

McNeely, M., et al. (2015). "Impacts of dance on non-motor symptoms, participation, and quality of life in Parkinson disease and healthy older adults." Maturitas 82(4): 336-341.

Mou, L., et al. (2019). "Open questions on the nature of Parkinson's disease: from triggers to spreading pathology." Journal of medical genetics: jmedgenet-2019-106210.

Oberman, L. M., et al. (2005). "EEG evidence for mirror neuron dysfunction in autism spectrum disorders." Cognitive brain research 24(2): 190-198.

San Luciano, M., et al. (2016). "Digitized spiral drawing: A possible biomarker for early Parkinson's disease." PloS one 11(10): e0162799.

Snaith, R., et al. (1986). "Grade Scores of the Montgomery—Åsberg Depression and the Clinical Anxiety Scales." The British journal of psychiatry 148(5): 599-601.

Tiwari, P. C. and R. Pal (2017). "The potential role of neuroinflammation and transcription factors in Parkinson disease." Dialogues in clinical neuroscience 19(1): 71.

Uitti, R. J., et al. (2005). "Parkinson disease: handedness predicts asymmetry." Neurology 64(11): 1925-1930.
